# Neuroprotective effects of low-dose G-CSF plus meloxicam in a rat model of anterior ischemic optic neuropathy

**DOI:** 10.1038/s41598-020-66977-9

**Published:** 2020-06-25

**Authors:** Pei-Kang Liu, Yao-Tseng Wen, Wei Lin, Kishan Kapupara, Minghong Tai, Rong-Kung Tsai

**Affiliations:** 1 Department of Ophthalmology, Kaohsiung Medical University Hospital, Kaohsiung Medical University, Kaohsiung, Taiwan; 2 Department of Ophthalmology, Yuan’s General Hospital, Kaohsiung, Taiwan; 3 Institute of Biomedical Sciences, National Sun Yat-Sen University, Kaohsiung, Taiwan; 4 School of Medicine, College of Medicine, Kaohsiung Medical University, Kaohsiung, Taiwan; 5 Institute of Eye Research, Hualien Tzu Chi Hospital, Buddhist Tzu Chi Medical Foundation, Hualien, Taiwan; 6 Department of Optometry, Da-Yeh University, Changhwa, Taiwan; 7 Center for Neuroscience, National Sun Yat-Sen University, Kaohsiung, Taiwan; 8 Graduate Program in Marine Biotechnology, National Sun Yat-Sen University, Kaohsiung, Taiwan; 9 Institute of Medical Sciences, Tzu Chi University, Hualien, Taiwan

**Keywords:** Neuroimmunology, Molecular medicine

## Abstract

Non-arteritic anterior ischemic optic neuropathy (NAION) causes a sudden loss of vision and lacks effective treatment. Granulocyte colony-stimulating factor (G-CSF) provides neuroprotection against the experimental optic nerve injuries but also induce leukocytosis upon typical administration. We found synergetic neuroprotective effects of meloxicam and low dose G-CSF without leukocytosis in a rat model of anterior ischemic optic neuropathy (rAION). The WBC counts in the low-dose G-CSF-plus meloxicam-treated group were similar to the sham-operated group. Combination treatment of low-dose G-CSF plus meloxicam preserved RGCs survival and visual function, reduced RGC apoptosis and the macrophages infiltration, and promote more M2 phenotype of macrophage/microglial transition than the low-dose GCSF treatment or the meloxicam treatment. Moreover, the combination treatment induced higher serine/threonine kinase 1 (Akt1) expression. The combination treatment of low-dose G-CSF plus meloxicam lessened the leukocytotic side effect and provided neuroprotective effects via Akt1 activation in the rAION model. This approach provides crucial preclinical information for the development of alternative therapy in AION.

## Introduction

Nonarteritic anterior ischemic optic neuropathy (NAION) is the most common acute optic neuropathy in people older than 50 years. The estimated mean annual incidence rates of NAION are 2.3–10.3 per 100,000 in the United States and 3.72 per 100,000 in Taiwan^1–3^. The debilitating consequences for patients with NAION include severe vision impairment and visual field defect. The primarily proposed pathogenesis of NAION is the transient nonperfusion or hypoperfusion of the optic nerve head and loss of retinal ganglion cells (RGCs) following ischemic insults. The histologic evidence in an early clinical case of human NAION demonstrated the presence of apoptotic cells in the RGC layer by 30 days after the event. This reaction eventually results in RGC death and vision loss^4^. Currently, no definite treatment for NAION is available. Halting the injury and loss of RGCs may, therefore, rescue the visual deterioration and be a potential treatment strategy.

Granulocyte colony-stimulating factor (G-CSF), a member of the cytokine family of growth factors, is a 19.6-kDa glycoprotein. It is commonly applied clinically to treat neutropenia^5^. G-CSF can induce the mobilization of CD34 + hematopoietic stem cells from the bone marrow into the peripheral blood. G-CSF is commonly used in clinics for stem cell mobilization and bone marrow reconstitution^6–8^. Our previous studies have demonstrated that G-CSF exerts neuroprotective effects on a rat model of anterior ischemic optic neuropathy (rAION). The protective effects of G-CSF on the rAION model are achieved through dual mechanisms of anti-inflammation and antiapoptosis^9,10^. The antiapoptotic effect of G-CSF occurs through the activation of a variety of intracellular signaling pathways and is mainly dependent on phosphatidylinositol-3 kinase (PI3K)/protein kinase B (AKT) activation^11,12^. Early intervention with G-CSF can induce M2 microglia/macrophage polarization, reduce the expression of proinflammatory cytokines, and stabilize the blood–optic nerve barrier (BOB) to reduce macrophage infiltration in the rAION model^10^. However, systemic treatment with G-CSF may result in leukocytosis and some side effects, such as arthralgia, bone pain, headache, fatigue, nausea, fever, chills, and myalgia^13^. Our previous study also demonstrated that subcutaneous administration of G-CSF (100 μg/kg/day) for five consecutive days could result in leukocytosis in rats^11^. Thus, developing a new approach to reducing the side effects of G-CSF treatment and maintaining the therapeutic effects in the rAION model may provide benefits.

Meloxicam belongs to the enolic acid group of nonsteroidal anti-inflammatory drugs (NSAIDs) and is clinically prescribed for treating certain types of arthritis, such as rheumatoid arthritis, for reducing inflammation and pain^14^. The possible mechanism of meloxicam is inhibition of migration of leukocytes and blocking cyclooxygenase (COX), the enzyme responsible for the first step in the synthesis of prostaglandins^15^. COX can convert arachidonic acid into prostaglandin H2, which is a mediator of inflammation. Meloxicam has been demonstrated to primarily inhibit COX-2, the cyclooxygenase isozyme, mainly in inflamed tissues with long-lasting anti-inflammatory and analgesic effects^16^. Preferential inhibition of COX-2 leads to high anti-inflammatory potency with lower ulcerogenicity in the stomach and higher tolerability than non-selective NSAIDs. Moreover, the metabolites of meloxicam are inactive^16^. Therefore, it has the potential to reduce inflammation in ischemic optic neuropathy. However, whether meloxicam can enhance the therapeutic effects of G-CSF treatment for AION remains unclear.

Macrophage/microglia has been shown to be polarizable and classifiable into M1 and M2 phenotype by expressed cytokine/chemokine profiles, surface markers, and biological functions. M1 phenotype is responsible for immunostimulation, including inflammation trigger, cell proliferation inhibition, which may cause tissue damage; M2 phenotype promotes immunosuppression and inhibits inflammation, which may improve cell proliferation and facilitate tissue repair in nervous tissues^17^. Activation of M2 phenotype macrophages, reduction in proinflammatory cytokine expression, and stabilization of the BOB are supposed to be three vital approaches to optic nerve protection in the rAION model^10,18^. Enhancement of anti-inflammatory actions through different mechanisms may serve as a potential strategy in the treatment of ischemic optic nerve injury. The purpose of this study was to investigate whether treatment with low-dose G-CSF plus meloxicam results in a synergistic effect of neuroprotection and reduction of the side effect of leukocytosis in the rAION model.

## Materials and methods

### Experimental animals

In this study, adult male Wistar rats weighing 150–180 g were used. The rats were purchased from the breeding colony of BioLASCO Co., Taiwan. Animal care and experimental procedures were performed in accordance with the Association for Research in Vision and Ophthalmology Statement for the Use of Animals in Ophthalmic and Vision Research. The Institutional Animal Care and Use Committee of Tzu Chi Medical Center approved all animal experiments.

### Study design

In the drug-induced leukocytosis study, 18 rats were equally divided into six groups including the sham-operated, the PBS-treated, the meloxicam-treated (0.125 mg/kg/day, Boehringer Ingelheim, Ingelheim am Rhein, Germany), the low-dose G-CSF-treated (50 μg/kg/day in 0.2 mL of saline, Takasaki Pharmaceutical Plant, Tokyo, Japan), high-dose G-CSF-treated (100 μg/kg/day in 0.2 mL of saline, Takasaki Pharmaceutical Plant), and low-dose G-CSF (50 μg/kg/day) plus meloxicam (0.125 mg/kg/day)-treated group. The meloxicam was administered via the oral route and the G-CSF via subcutaneous injection.

In the therapeutic evaluation, after successful induction of AION in the rats, 120 rats were equally divided into four groups. Treatment with subcutaneous injection of low-dose G-CSF only once daily, meloxicam only, low-dose G-CSF plus meloxicam, and phosphate-buffered saline (PBS, serving as control; 0.2 mL) alone immediately after the rAION procedure for a total of 5 consecutive days. Another 30 rats received sham laser treatment without a photosensitizing agent to serve as normal controls. The number of rats used in this study is summarized in Fig. S1. All the rats tolerated this treatment and survived until the end of the procedure.

### Peripheral WBC count

After the drug administration, 2–3 mL of circulating blood was drawn by cardiac puncture from the rats one week after rAION induction. WBC counts were obtained.

### rAION induction

The method of rAION induction was the same as that used in our previous report^9,10^. Before general anesthesia, the rats were treated with Alcaine and Mydrin-P eye drops for topical anesthesia and pupil dilation, respectively. The rat was administrated by intramuscular injections of a mixture of ketamine (40 mg/kg body weight) and xylazine (4 mg/kg body weight; Sigma, St. Louis, MO, USA) for general anesthesia. Subsequently, 2.5 mM rose bengal in PBS (1 ml/kg animal weight) was intravenously administered. After rose bengal injection, the optic disc was immediately exposed to an argon green laser system (MC-500 Multi-color laser, Nidek Co., Ltd, Tokyo, Japan, setting: 532 nm wavelength, 500 μm size and 80 mW power) for 12 1-s pulses^19,20^. A laser fundus lens (Ocular instruments Inc.) was used to focus the laser on the optic disc. (Supplement Fig. 2) Tobradex eye ointment was applied after the procedure, and the rats were monitored until complete recovery was observed.

### Retrograde labeling of RGCs with FluoroGold and morphometry of the RGCs

The detailed methods and protocol of FluoroGold labeling have been described in our previous reports^9,18^. Briefly, the retinas were examined at distances of 1 mm from the center of the optic nerve for RGC counting to obtain the RGC density in the central retina. We calculated eight randomly selected areas in the central retina; the total area counted was about 13.5 mm^2^. The densities of RGC were averaged for each retina (n = 12 rats per group).

### Flash visual evoked potentials (FVEP)

The detailed FVEP recording method has been described in our previous studies^9,10^. For electrode placement, the sagittal region of the skull was opened in the rats. The 4 mm screw implants were pass through the skull approximate 1.5 mm and were placed at the frontal cortex and the primary visual cortex region of both hemispheres using stereotaxic coordinates. A visual electrodiagnostic system (Diagnosys LLC, Lowell, MA, USA) was used to measure the FVEP. The number of sweeps per average was 64 for each rat. A comparison of the amplitude of the P1-N2 wave in each group was made to evaluate visual function (n = 12 rats per group).

### Optic nerve and retinal sample preparation

The rats were euthanized after four weeks; the eyes were enucleated along with the optic nerve (approximately 5 mm in length). The samples were fixed in 4% paraformaldehyde and were cryoprotected by 30% sucrose; the samples were stored at 4 °C until they settled at the bottom of the tubes. Sections of 20 μm were obtained using a cryostat.

### TUNEL assay

To ensure the use of equivalent fields for comparison, all the paraffin or frozen sections of the retina were prepared with retinas at 1 to 2 mm distance from the ON head. TUNEL assay was used to detect apoptotic cells in the ganglion cell layer (GCL). This assay was performed according to the manufacturer’s procedure (DeadEnd Fluorometric TUNEL System; Promega Corporation, Madison, WI, USA). The TdT-dUTP terminal nick-end labeling-positive cells in the GCL of each sample were calculated in 10 high-powered fields (HPF, 400×), and an average from three sections per retina was used for further comparison (n = 6 rats per group).

### Immunohistochemical staining for phagocytic macrophage/microglia detection

ED-1 is a marker for phagocytic macrophages and microglia. The longitudinal section of the optic nerve was blocked with 5% FBS for one hour at room temperature. The section was labeled with an ED1 primary antibody diluted in the dilution buffer (2% BSA, 1 × PBS (pH 7.2), and 0.3% Triton X-100; 1: 200) overnight at 4 °C. The section was incubated with Goat anti-mouse Alexa 488 (0.3% Triton X-100 and 1 × PBS (pH 7.2); 1: 500) for one hour at room temperature and counterstained with DAPI (0.3% Triton X-100 and 1 × PBS (pH 7.2); 1: 500). The image was acquired by using appropriate filter sets in a fluorescence microscope at × 100 magnification. ED-1+ cell counting was conducted by ImageMaster 2 Platinum software.

### Quantitative reverse transcription polymerase chain reaction

The markers of M2 macrophages (Arg 1, CD206, and Fizz1) were evaluated^10,18^. Tissue RNA was extracted from optic nerve lysates obtained using the sonication method (130 W, 30% amplitude, 5 pulses for each sample, each pulse consists of 5 seconds ON and 2 seconds OFF cycle) with a Qiagen RNeasy Mini Kit (Hilden, Nordrhein-Westfalen, Germany). All the RNA samples were reverse transcribed at 42 °C for 30 minutes by using a high-capacity cDNA reverse transcription kit (Applied Biosystems, Foster City, CA, USA). qRT-PCR was then conducted on an AB PRISM 7300 Sequence Detection System (Applied Biosystems) with the QuantiTect SYBR green qRT-PCR kit (Qiagen). Expression levels of CypA mRNA were used for normalization to measure the expression levels of each target gene. The calculated data are presented as the mean relative expression levels ± standard deviation (SD). The primers used in this study for target gene amplification were listed in Table S1.

### Western blotting analysis

The optic nerve samples were collected on day seven after rAION induction in each group. The protein extracts of the optic nerve were separated by using a 4–12% NuPAGE Bis-Tris gel (Invitrogen, Carlsbad, CA, USA). The separated proteins were transferred onto polyvinylidene difluoride membranes. The membranes were blocked by using 5% nonfat milk in Tris buffer saline/Tween-20 solution containing 20 mM Tris-HCl (pH 7.5), 0.5 M NaCl, and 0.5% Tween-20. Subsequently, the membranes were blotted with mouse anti-p-Akt1 antibody (Abcam, Cambridge, MA, USA) followed by goat anti-mouse HRP. The developing reaction was performed by using an enhanced chemiluminescent substrate (Perkin-Elmer Life Science, Boston, MA, USA), and the relative intensities of the bands were measured by using an image analysis system (Amersham Biosciences, Uppsala, Sweden).

### Statistical analysis

All data are represented as Mean ± Standard deviation. We performed statistical analysis with SPSS commercial software (Chicago, IL, USA, USA). Mann–Whitney U test was used to evaluate the differences between each group individually. Statistical significance was set at p < 0.05. All measurements in this study were performed in a masked manner.

## Results

### Evaluation of leukocytosis after treatment

**Figure 1:**
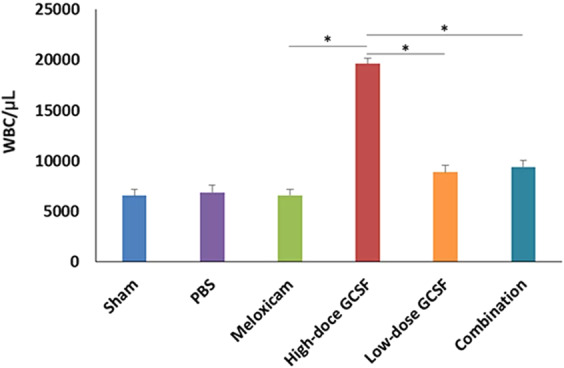
Analysis of WBC counts in the sham-operated rats, PBS-treated rats, meloxicam-treated rats, high-dose G-CSF-treated rats, low-dose G-CSF-treated rats, and low-dose G-CSF-plus-meloxicam-treated rats. Treatment with meloxicam, low-dose G-CSF, and low-dose G-CSF plus meloxicam did not induce leukocytosis in the rats, but treatment with high-dose G-CSF induced leukocytosis after a 5-day treatment. *p < 0.05.

After 5-day treatment with high-dose G-CSF, low-dose G-CSF, meloxicam, and low-dose G-CSF plus meloxicam, the WBC counts were 6562 ± 662/μL, 6889 ± 693/μL, 6613 ± 557/μL, 19,633 ± 531/μL, 8897 ± 712/μL, and 9415 ± 657/μL in the sham, PBS-treated, meloxicam-treated, high-dose G-CSF-treated, low-dose G-CSF-treated, and low-dose G-CSF-plus-meloxicam-treated groups, respectively. The WBC counts increased by 2.99-fold in the high-dose G-CSF-treated group compared with the sham group (p = 0.0011), which indicates the leukocytosis effects of high-dose G-CSF. Treatments with meloxicam, low-dose G-CSF, and low-dose G-CSF plus meloxicam did not induce leukocytosis in the animals (Fig. 1). Therefore, we used low-dose G-CSF instead of high-dose G-CSF with or without meloxicam in the following experiments.

### Combined treatment preserved more visual function than other single treatments

**Figure 2:**
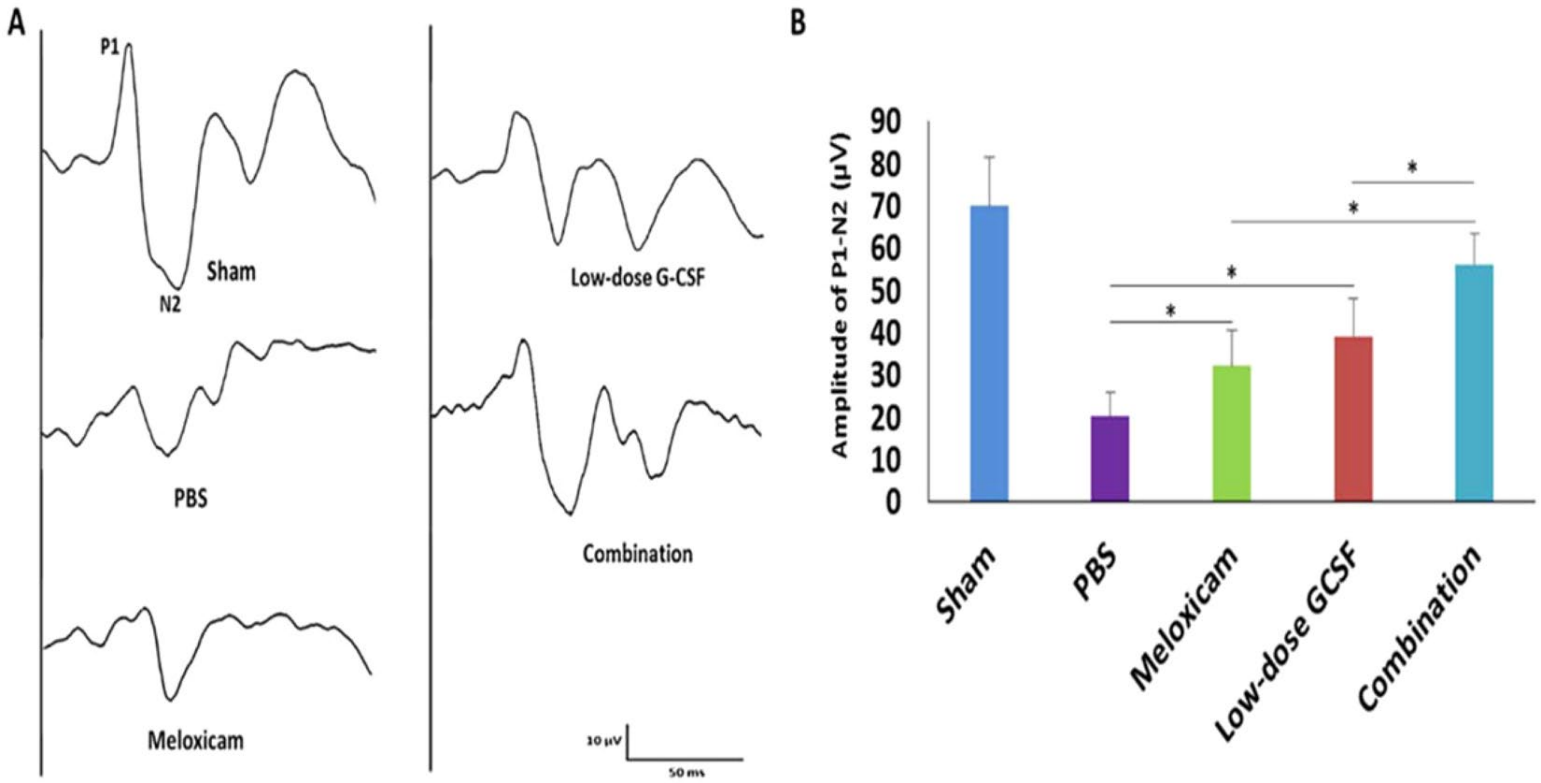
Evaluation of the visual function by using FVEPs in the rAION model. (**A**) Representative FVEP tracings at four weeks after rAION induction in the sham group, PBS-treated group, meloxicam-treated group, low-dose G-CSF-treated group, and low-dose G-CSF-plus-meloxicam-treated group. (**B**) Bar charts demonstrate the P1-N2 amplitude. The values of amplitude are expressed as mean ± SD in each group (n = 12 in each group). The amplitudes of the P1-N2 waves in the combination-treated group were significantly higher than meloxicam-treated and low-dose G-CSF-treated groups, respectively. *p < 0.05.

We performed FVEP to evaluate the visual function. The FVEP of the sham, PBS-, meloxicam-, low-dose G-CSF-, and the combination-treated group were recorded (Fig. 2A). The P1 latency did not exhibit a significant difference among the groups in the FVEP tests. The amplitudes of the P1-N2 waves in the combination-treated group were significantly higher than the meloxicam-treated group and low-dose G-CSF-treated group (56.3 ± 7.4 vs. 31.8 ± 8.5 and 39.2 ± 8.8 μv; p = 0.011 and 0.011, respectively; Fig. 2B). In addition, the amplitudes of the P1-N2 waves were significantly higher in the meloxicam-treated group and low-dose G-CSF-treated group compared with PBS-treated group (31.8 ± 8.5 and 39.2 ± 8.8 vs. 20.3 ± 6.1 μv, p = 0.021 and 0.021, respectively; Fig. 2B).

### The combined treatment protects RGCs from apoptosis and increases survival

**Figure 3:**
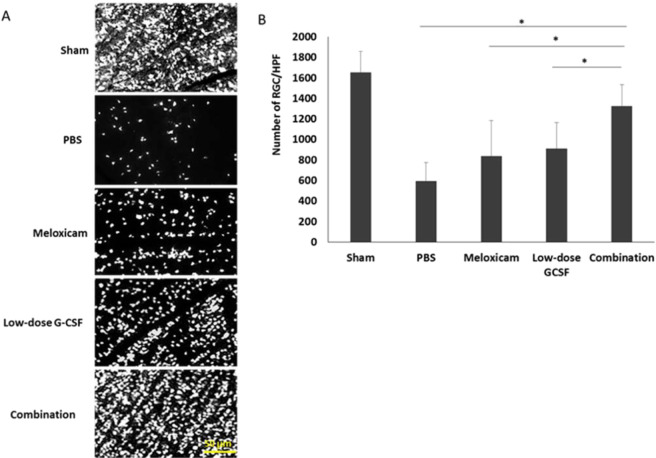
Survival of RGCs in rAION-induced rats with PBS treatment, meloxicam treatment, G-CSF treatment, and G-CSF plus meloxicam treatment at 28 days after rAION induction. (**A**) A representative of flat-mounted central retinas and the morphometry of RGCs in each group through FluoroGold retrograde labeling at four weeks after rAION induction. (**B**) RGC density in the central retina in each group. Data are expressed as mean ± SD for each group (n = 12). The number of RGCs in the combination-treated group was 1.58- and 1.45-fold higher than in the meloxicam-treated and low-dose G-CSF-treated groups, respectively. *p < 0.05.

**Figure 4:**
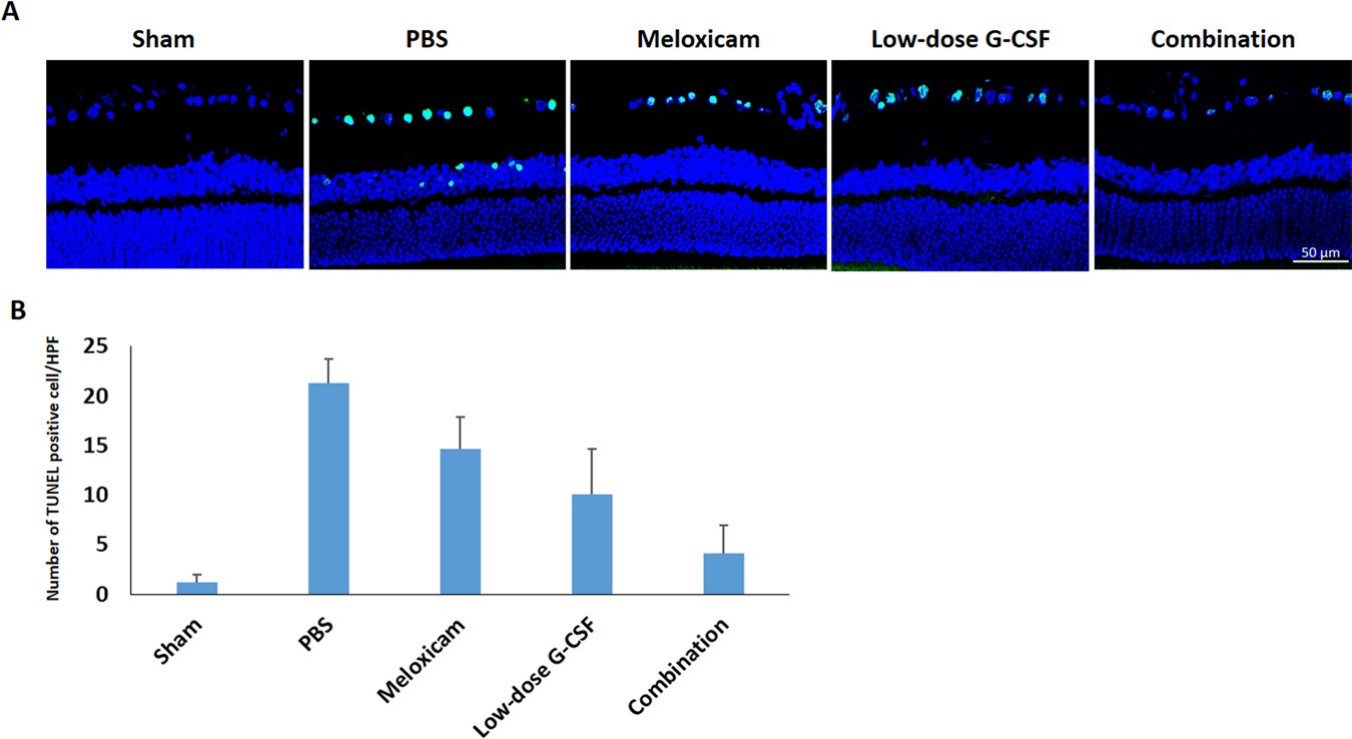
Analysis of RGC apoptosis in the RGC layer through TUNEL assay at four weeks after rAION induction. (**A**) Representative images of double-stained apoptotic cells in the RGC layers in each group. The apoptotic cells (TUNEL-positive cells) in green were stained with TUNEL staining, and the nuclei of the RGCs in blue were labeled with DAPI staining. (**B**) Quantification of TUNEL-positive cells per high-power field. Data are expressed as mean ± SD for each group (n = 6). Treatment with low-dose G-CSF plus meloxicam significantly reduced the number of apoptotic RGC by 3.6- and 2.5-fold compared with the meloxicam-treated and low-dose G-CSF-treated groups, respectively. *p < 0.05.

In contrast to treatment with PBS, both the low-dose G-CSF- and meloxicam-treatment groups preserved a higher density of RGCs in the central retinas (Fig. 3A). Four weeks after rAION induction, the RGC density in the central retinas in the sham, PBS-, meloxicam-, low-dose G-CSF-, and combination-treated group was 1652.3 ± 210.3/mm^2^, 587.8 ± 187.3/mm^2^, 840.1 ± 344.7/mm^2^, 911.6 ± 253.5/mm^2^, and 1321.3 ± 126.5/mm^2^ respectively (Fig. 3B). The number of RGCs in the combination-treated group was 1.58- and 1.45-folds higher than the meloxicam-treated group (p = 0.021) and low-dose G-CSF-treated group (p = 0.021), respectively. Apoptotic cells (TUNEL + cells) in RGC layer in sham, PBS-, meloxicam-, low-dose G-CSF-, and the combination-treated group was 1.2 ± 0.8/HPF, 21.3 ± 2.4/HPF, 14.7 ± 3.2/HPF, 10.1 ± 4.6/HPF and, 4.1 ± 2.9/HPF, respectively (Fig. 4A,B). Treatment with low-dose G-CSF plus meloxicam significantly reduced the number of apoptotic RGCs by 3.6- and 2.5-folds (p = 0.018 and 0.021, respectively) compared with treatment with meloxicam and low-dose G-CSF.

### Combined treatment reduced extrinsic macrophage infiltration and increased the level of M2 phenotypic markers

**Figure 5:**
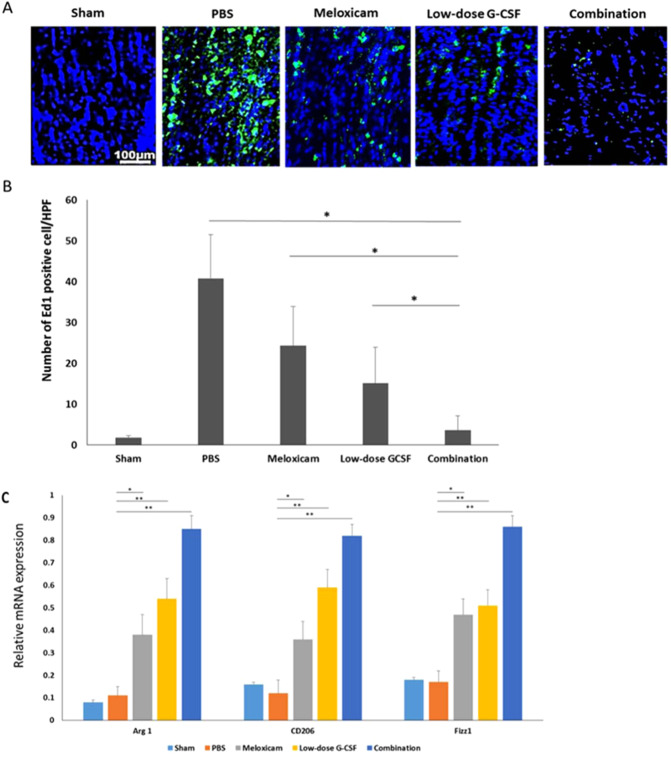
Immunohistochemistry (IHC) of ED1 in the optic nerve at four weeks after rAION induction for evaluating the inflammatory infiltration of macrophages. (**A**) Representative images of ED1 staining in the longitudinal sections of the optic nerve. The ED1-positive cells in green were stained with FITC, and the nuclei in blue were labeled with DAPI. (**B**) Quantification of ED1-positive cells per high-power field. Data are expressed as mean ± SD in each group (n = 6). Macrophage recruitment was decreased by 6.75- and 4.1-fold in the combination-treated group compared with the meloxicam-treated and low-dose G-CSF-treated groups, respectively (**C**) Evaluation of M2 macrophage polarization at four weeks after rAION induction. Relative mRNA expression levels of the markers of M2 macrophages in the optic nerve are shown as histograms. Each value was normalized to CypA. The expression levels of Arg 1, CD206, and Fizz1 (markers of M2 macrophages) increased after treatment with low-dose G-CSF plus meloxicam compared with treatment with PBS-treated group, meloxicam alone, and low-dose G-CSF alone, respectively. *p < 0.05, **p < 0.01.

Combination treatment synergistically reduced the number of ED1-positive cells in the rAION model (Fig. 5A). The number of ED1-positive cells/HPF in sham, PBS-, meloxicam-, low-dose G-CSF-, and the combination-treated group was 1.8 ± 0.5, 40.8 ± 10.7, 24.3 ± 9.6, 15.1 ± 8.9, and 3.6 ± 3.5, respectively (Fig. 5B). Macrophage recruitment decreased by 6.75- and 4.1-folds in the combination-treated group compared with the meloxicam-treated (p = 0.021) and low-dose G-CSF-treated group (p = 0.032), respectively. Further, the qRT-PCR analysis demonstrated that the mRNA levels of Arg 1, CD206, and Fizz1 (M2 phenotypic markers) increased after treatment with meloxicam, low-dose G-CSF, and low-dose G-CSF plus meloxicam after rAION induction compared with PBS-treated group. In addition, the combination treatment exerted synergistic effects on the increased expression of Arg1, CD206, Fizz; (p = 0.005) in the rAION model (Fig. 5C).

### Combination treatment induced more Akt1 activation than other single treatments

**Figure 6:**
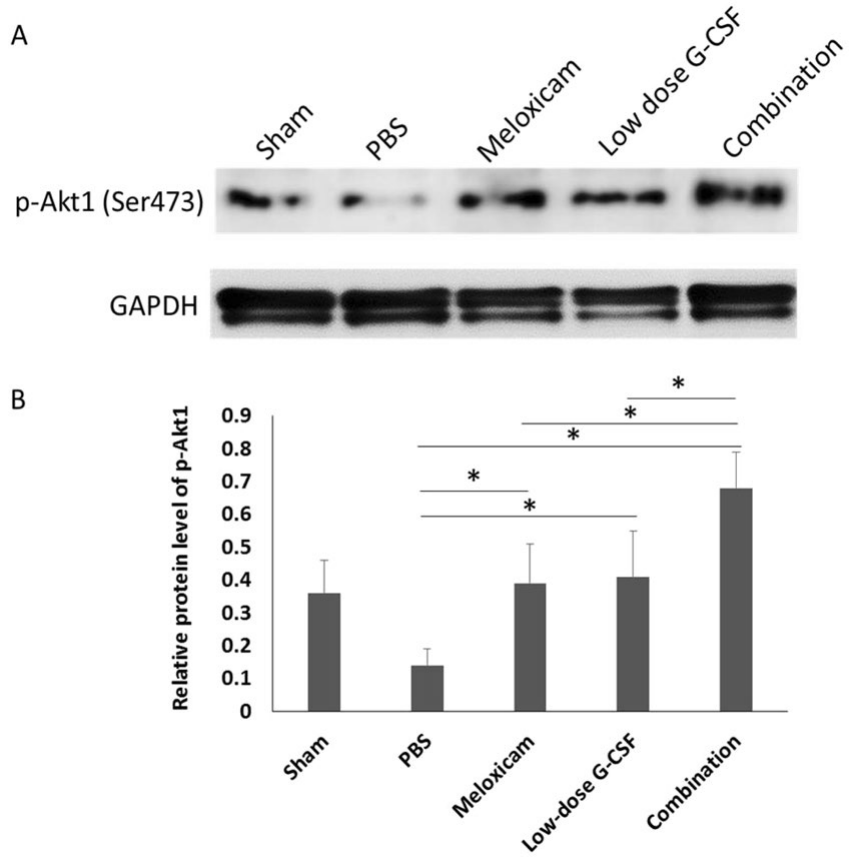
Immunoblots of the optic nerve. (**A**) Analysis of p-Akt1 expression by using Western blotting. (**B**) Quantification of the protein bands of p-Akt1. Each value was normalized to GAPDH. Data are expressed as mean ± SD in each group (n = 6 in each group). The combination treatment induced higher p-Akt1 expression than treatment with meloxicam or low-dose G-CSF in the rAION model. *p < 0.05.

To reveal the synergetic effects of the combination treatment, the expression level of p-Akt1 was assessed at day seven after AION induction to determine if combination treatment had an enhanced effect on p-Akt1 expression compared to meloxicam or low dose G-CSF (Fig. 6A,B). The levels of p-Akt1 in the meloxicam-treated group (p = 0.018), low-dose G-CSF-treated group (p = 0.021), and combination-treated group (p = 0.011) were 2.78-, 2.93-, and 4.86-fold higher than PBS-treated group, respectively. Besides, the combination treatment induced higher p-Akt1 expression than treatment with meloxicam (p = 0.021) or low-dose G-CSF (p = 0.021) in the rAION model.

## Discussions

Consecutive high-dose G-CSF (100 μg/kg/day) injection for five days commonly triggered leukocytosis in rats, but this undesired effect was absent with low-dose G-CSF (50 μg/kg/day) treatment. The administration of low-dose G-CSF alone or meloxicam alone yielded preservation of visual function, better RGCs survival, reduction of macrophage infiltration, and more M2 macrophage/microglia polarization after rAION insults. Notably, the neuroprotective effects of the combination treatment worked synergistically. In addition, the combination treatment induced more p-Akt1 expression than meloxicam or low-dose G-CSF treatment alone in the current model.

Based on our past work, we concluded that subcutaneous injection of high-dose G-CSF provided neuroprotection in both rAION and optic nerve crush (ONC) models^9–12^. We also found that leukocytosis was induced by this therapy in the rat ONC model^11^. Moreover, this therapy has detrimental effects on patients. Commonly encountered side effects after G-CSF use comprise arthralgia, bone pain, fatigue, nausea, fever, chills, headache, and myalgia^13^. Thus, for clinical applications, reducing the undesired effects induced by G-CSF therapy is essential for ophthalmic diseases. In this study, we found that consecutively treating normal Wistar rats with high-dose G-CSF (100 μg/kg/day) via subcutaneous injection would induce leukocytosis. By contrast, neither low-dose G-CSF (50 μg/kg/day) treatment nor meloxicam treatment would lead to the undesired effect. As expected, the combination treatment did not induce leukocytosis compared with normal rats. Furthermore, the addition of meloxicam to G-CSF treatment may reduce the discomfort caused by the side effects of G-CSF because meloxicam is a strong pain reliever. From the literature review, meloxicam possesses low toxicity to gastrointestinal tract mucosa and trustworthy efficacy against inflammation and pain^21–24^. Therefore, treatment with a combination of G-CSF and meloxicam is a reasonable approach for rAION.

The neuroprotective role of meloxicam on the rAION model was first disclosed in this study. Surprisingly, meloxicam revealed comparable neuroprotective effects to low-dose G-CSF regarding antiapoptosis, inhibition of macrophage infiltration, and macrophage M2 polarization in this model. Meloxicam breaks the inflammatory cascade via inhibition of MAPK and p53 signaling pathway and owns antiapoptotic effects evidenced by alleviating oxidative stress, avoiding mitochondrial dysfunction, and reducing endoplasmic reticulum stress response *in vitro*^25,26^. A recent study reported that meloxicam’s neuroprotective effects maintained cell survival via the upregulation of the PI3K/Akt pathway instead of COX-2 inhibition^26^. In this study, we further disclosed that meloxicam induced Akt1 activation on day seven post-infarct. Couples of previous studies have indicated that the upregulation of the PI3K/AKT signaling pathway benefits the survival of injured RGCs^27–29^. In our previous study, we also demonstrated that the apoptosis of RGCs following optic nerve crush could be antagonized by G-CSF via PI3K/AKT-signaling pathway^12^. Taken together, we consider that meloxicam can maintain Akt1 activation to prevent RGC death after optic nerve damage. Remarkably, treatment with a combination of meloxicam plus low-dose G-CSF induced higher Ak1 activation than treatment with meloxicam only or low-dose G-CSF only in the rAION model. Therefore, we believe that combination treatment with meloxicam and low-dose G-CSF affords better protection of RGCs in the rAION model via antiapoptosis of RGCs and activation of the Akt1 signaling pathway.

After rAION induction, ED1 + phagocytes, including monocytes/macrophages of hematopoietic origin and microglia, are recruited into the optic nerves. The hematogenous ED1 + cells found at the optic nerve following rAION induction implicates disrupted BOB^10,30^. We found that ED1 + macrophage/microglial buildup at the optic nerve lesion site was alleviated in the meloxicam-treated and low-dose G-CSF-treated groups. The effect of attenuation was enhanced when the combination treatment of low-dose G-CSF plus meloxicam was used. Our recent study, therefore, implies that the rAION-injured optic nerves may be protected by the anti-inflammatory effects of immediate administration of meloxicam or G-CSF, and combination treatment with G-CSF plus meloxicam exerts the synergistic effect of reduction in macrophage recruitment. Some studies have demonstrated that treatment with G-CSF is able to reduce the breakdown of blood-brain barrier (BBB), brain edema, and macrophage infiltration in experimental brain injury models^30–33^. Also, our previous report revealed that protection from BOB disruption reduces macrophage infiltration into the optic nerve lesion site^10^. We believe that the possible mechanism of BOB disruption is associated with the modulation of the Akt signaling pathway in the rAION model. Our study also noted that Akt1 was activated by treatment with meloxicam or G-CSF alone in the rAION model. Therefore, we suggest that Akt1 activation may play a crucial role in preventing BOB breakdown after optic nerve infarct.

Macrophage/microglia has plasticity, and one phenotype can convert into another phenotype when driven by cytokines in different microenvironments^34^. We ever reported that phenotype switch of macrophages from pro-inflammatory (M1) to anti-inflammatory (M2) prevent the cytokine-induced optic nerve injuries by reducing the pro-inflammatory cytokine expression and free radicals (e.g., IL-6, IL-1β, TNF-α, and iNOS) during the inflammatory responses of optic nerve injury^10,18^. Intriguingly, our findings showed that meloxicam, a COX-2 inhibitor, alone was able to activate M2 polarization on the injured optic nerve, and the protective phenomenon was further magnified by simultaneous G-CSF and meloxicam administration.

Hofer *et al*. reported that meloxicam has hematopoiesis-modulating action and can increase serum G-CSF levels in sublethally gamma-irradiated animals^35^. Enhancement of neuroprotective effects and M2 polarization from the combination treatment may be due to the increase in endogenous G-CSF after meloxicam administration. Also, we found that treatment with meloxicam alone or G-CSF alone activated Akt1 7 days after rAION induction. Since Akt downregulation will abolish the upregulation of M2 genes, Akt activation plays an important role in M2 macrophage differentiation^36^. Liu *et al*. demonstrated that TIPE2 activates the PI3K-AKT signaling pathway and subsequently promotes M2 polarization^37^. Furthermore, recent studies have reported that Akt1 and Akt2 kinase isoforms are critical to modulation of macrophage polarization and Akt1 ablation shifts macrophage towards M1 status^38^. Taken together, we conclude that Akt1 upregulation may be crucial to promote M2 polarization in the rAION model.

In conclusion, addressing anti-inflammatory therapy through different mechanisms may provide a potential approach to the management of ischemic optic nerve injury. Combination therapy of G-CSF plus meloxicam can reduce the leukocytosis side effects, prevent postinjury RGCs apoptosis, preserve the visual function, halt the recruitment of macrophages to the optic nerve, and promote the M2 phenotype transition in macrophages. The neuroprotective effects of G-CSF and meloxicam worked synergistically. However, additional works are mandatory to more clearly elucidate the underlying mechanisms of the interaction between G-CSF and meloxicam.

## Supplementary information

Supplementary information
