# Author Correction: Neuroprotective effects of low-dose G-CSF plus meloxicam in a rat model of anterior ischemic optic neuropathy

**DOI:** 10.1038/s41598-021-97922-z

**Published:** 2021-09-14

**Authors:** Pei-Kang Liu, Yao-Tseng Wen, Wei Lin, Kishan Kapupara, Minghong Tai, Rong-Kung Tsai

**Affiliations:** 1 Department of Ophthalmology, Kaohsiung Medical University Hospital, Kaohsiung Medical University, Kaohsiung, Taiwan; 2 Department of Ophthalmology, Yuan’s General Hospital, Kaohsiung, Taiwan; 3 Institute of Biomedical Sciences, National Sun Yat-Sen University, Kaohsiung, Taiwan; 4 School of Medicine, College of Medicine, Kaohsiung Medical University, Kaohsiung, Taiwan; 5 Institute of Eye Research, Hualien Tzu Chi Hospital, Buddhist Tzu Chi Medical Foundation, Hualien, Taiwan; 6 Department of Optometry, Da-Yeh University, Changhwa, Taiwan; 7 Center for Neuroscience, National Sun Yat-Sen University, Kaohsiung, Taiwan; 8 Graduate Program in Marine Biotechnology, National Sun Yat-Sen University, Kaohsiung, Taiwan; 9 Institute of Medical Sciences, Tzu Chi University, Hualien, Taiwan

Correction to: *Scientific Reports* 10.1038/s41598-020-66977-9, published online 25 June 2020

The original version of this Article contained an error in Figure 4 where the number of TUNEL-positive cells in Figure 4A did not match the number of cells in Figure 4B. The original Figure 4 and accompanying legend appear below.

**Figure 4:**
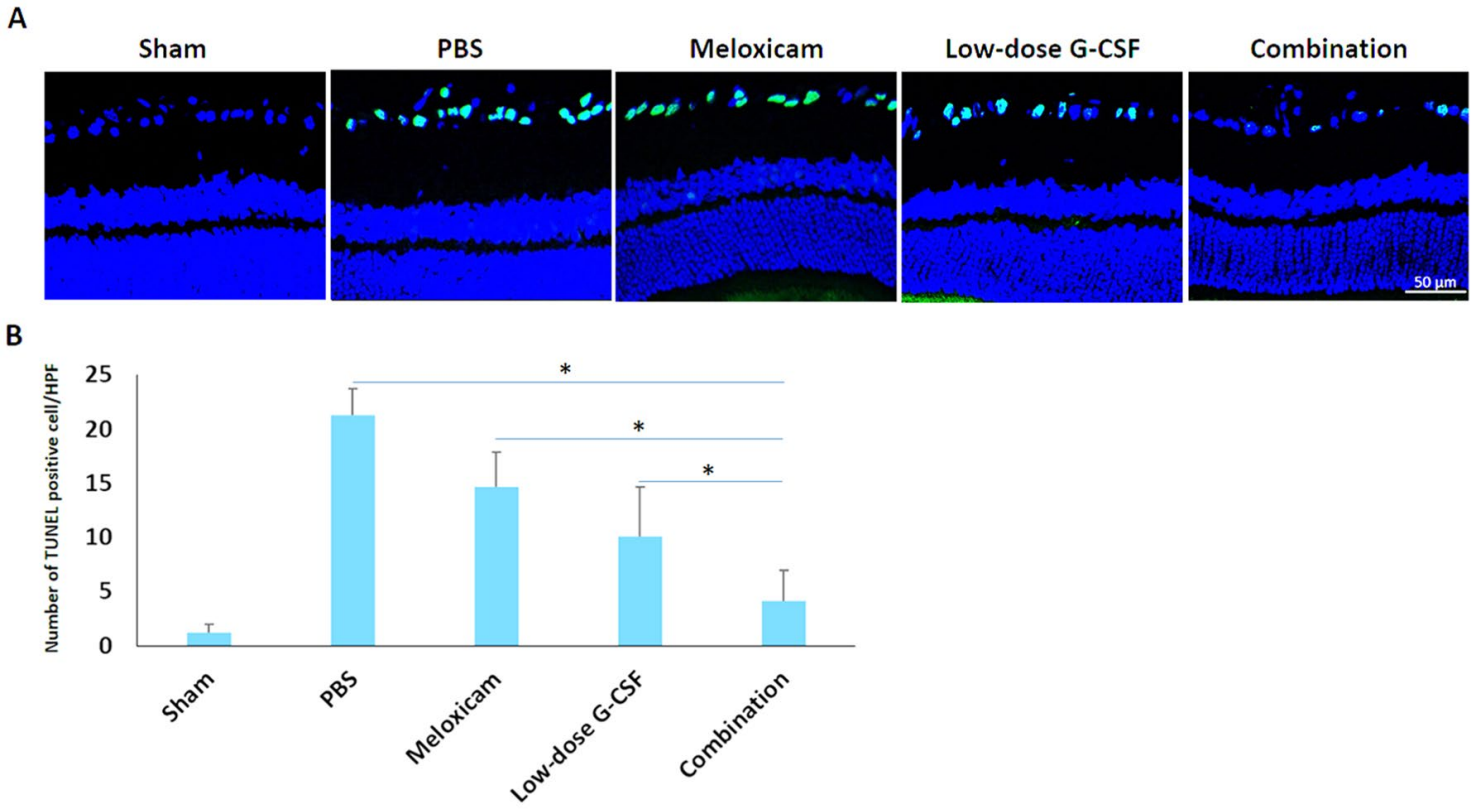
Analysis of RGC apoptosis in the RGC layer through TUNEL assay at four weeks after rAION induction. (**A**) Representative images of double-stained apoptotic cells in the RGC layers in each group. The apoptotic cells (TUNEL-positive cells) in green were stained with TUNEL staining, and the nuclei of the RGCs in blue were labeled with DAPI staining. (**B**) Quantification of TUNEL-positive cells per high-power field. Data are expressed as mean ± SD for each group (n = 6). Treatment with low-dose G-CSF plus meloxicam significantly reduced the number of apoptotic RGC by 3.6- and 2.5-fold compared with the meloxicam-treated and low-dose G-CSF-treated groups, respectively. *p < 0.05.

The original Article has been corrected.

