# Supplementary material for: Neuroprotective effects of low-dose G-CSF plus meloxicam in a rat model of anterior ischemic optic neuropathy
Source: Sci Rep. 2020 Jun 25;10:10351. doi: 10.1038/s41598-020-66977-9 (PMC7316837; doi:10.1038/s41598-020-66977-9)

**Neuroprotective effects of low-dose G-CSF plus meloxicam in a rat model of anterior ischemic optic neuropathy**

Pei-Kang Liu^1,2,3,4,^* ,Yao-Tseng Wen^5,^*, Wei Lin^6^ , Kishan Kapupara^5^, Ming-Hong Tai^3,7,8^ ,Rong-Kung Tsai^5,9^

^1^Department of Ophthalmology, Kaohsiung Medical University Hospital, Kaohsiung Medical University, Kaohsiung, Taiwan.

^2^Department of Ophthalmology, Yuan’s General Hospital, Kaohsiung, Taiwan

^3^Institute of Biomedical Sciences, National Sun Yat-Sen University, Kaohsiung, Taiwan

^4^School of Medicine, College of Medicine, Kaohsiung Medical University, Kaohsiung, Taiwan.

^5^Institute of Eye Research, Hualien Tzu Chi Hospital, Buddhist Tzu Chi Medical Foundation, Hualien, Taiwan

^6^ Department of Optometry, Da-Yeh University, Changhwa, Taiwan.

^7^ Center for Neuroscience, National Sun Yat-Sen University, Kaohsiung, Taiwan

^8^Graduate Program in Marine Biotechnology, National Sun Yat-Sen University, Kaohsiung, Taiwan

^9^Institute of Medical Sciences, Tzu Chi University, Hualien, Taiwan

*P-K-L and Y-T-W have equal contribution to this study.

**Corresponding author:**

Rong-Kung Tsai MD, PhD

Institute of Eye Research, Hualien Tzu Chi Hospital, Buddhist Tzu Chi Medical Foundation; Tzu Chi University, Hualien, Taiwan

Email: [rktsai@tzuchi.com.tw](mailto:rktsai@tzuchi.com.tw).

Ming-Hong Tai, Ph.D.

Institute of Biomedical Sciences, National Sun Yat Sen University, No.70 Lienhai Road, Kaohsiung, Taiwan

Email: [minghongtai@gmail.com](mailto:minghongtai@gmail.com)


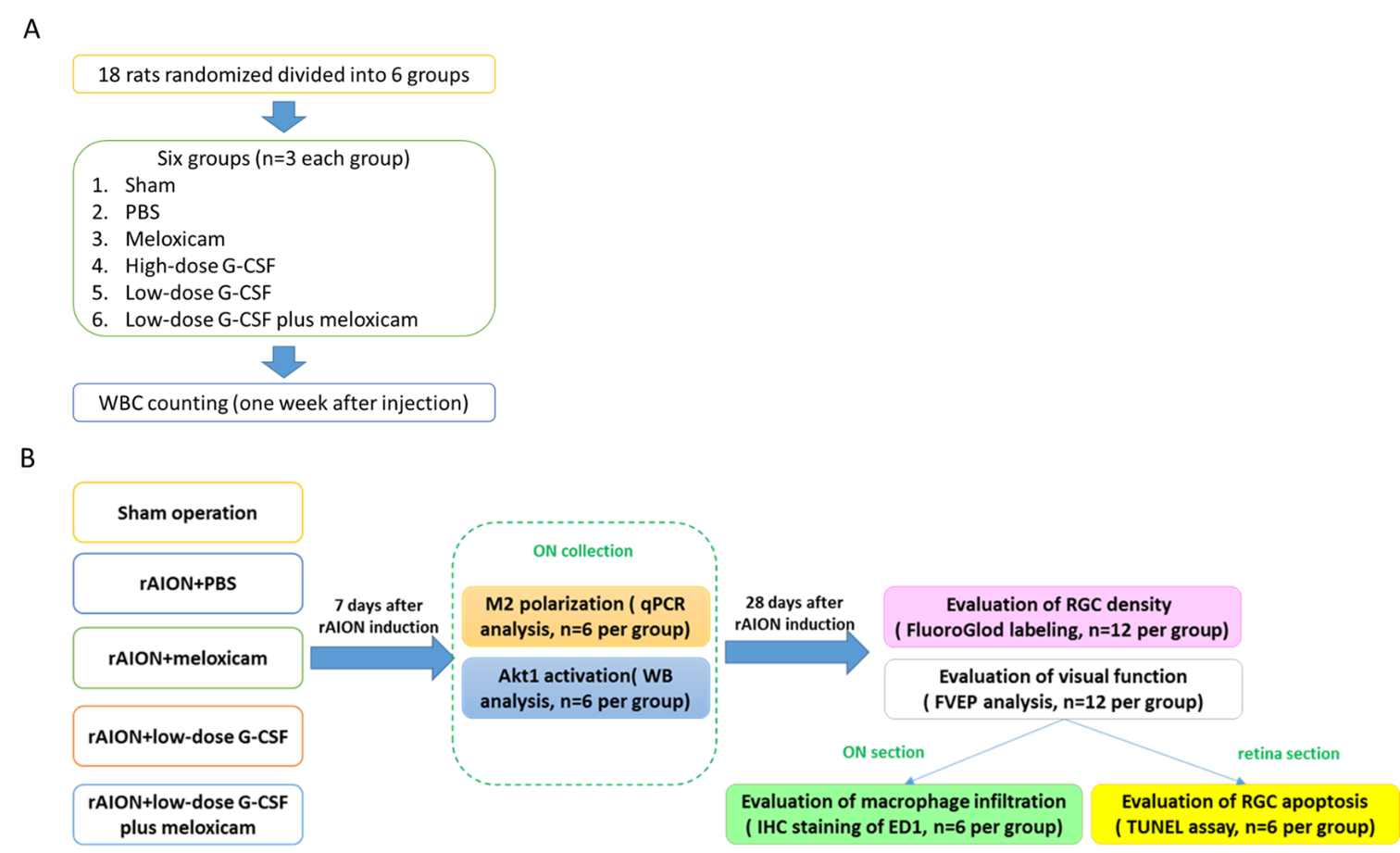


**Figure S1**. Summary of the study design to investigate the side effects and therapeutic effects of treatment with a combination of low-dose G-CSF plus meloxicam in an rAION model. (Dosage: meloxicam :0.125 mg/kg/day; low-dose G-CSF: 50 μg/kg/day in 0.2 mL of saline; high-dose G-CSF: 100 μg/kg/day in 0.2 mL of saline, G-CSF plus meloxicam: G-CSF 50 μg/kg/day plus meloxicam 0.125 mg/kg/day. The meloxicam was administered via the oral route and the G-CSF via subcutaneous injection.)


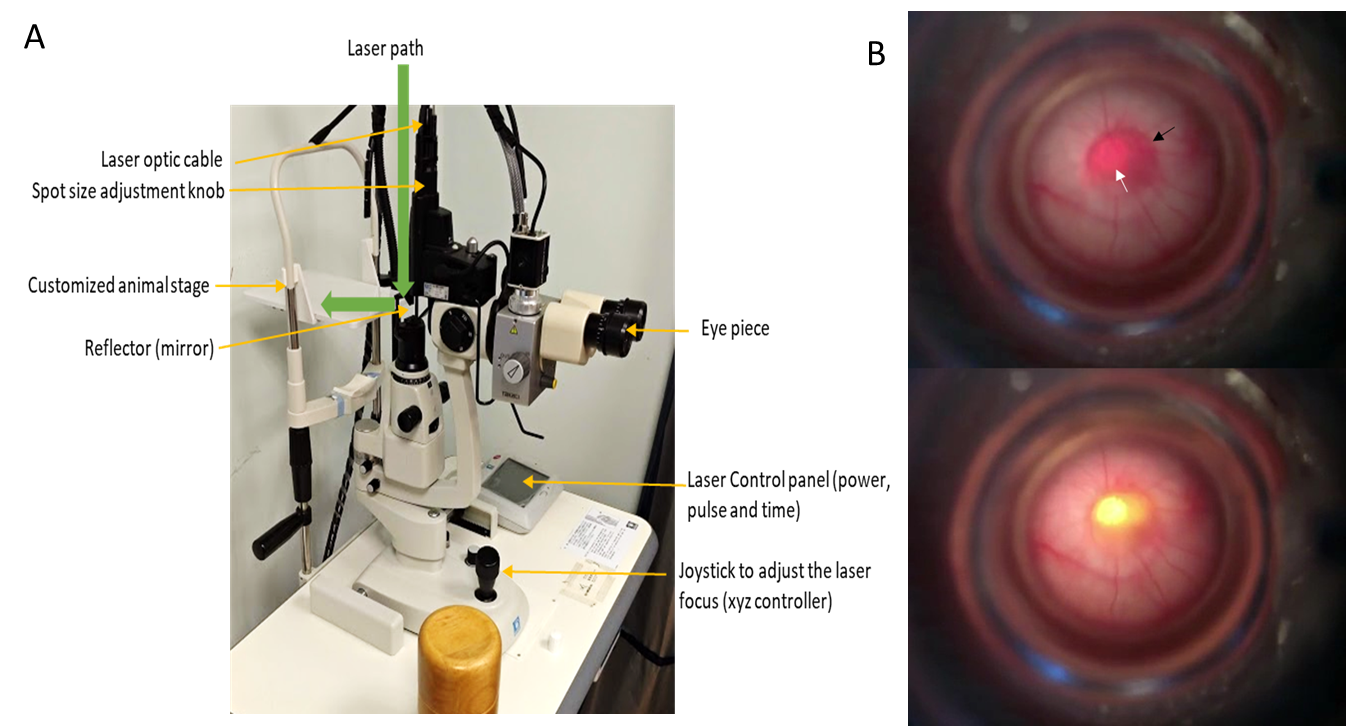


**Figure S2**. Setup for the generation of the rAION model in rats. **a**) a green laser-powered slit lamp ophthalmoscope for photoactivation of rose Bengal. **b**) above panel shows optic nerve head (black arrow) with a red pointer of 500 microns (white arrow). The pointer allows the operator to make sure that the green laser illuminates the desired region. Below panel showing the yellow spot is the photoactivation of rose Bengal by a green laser.

**Table S1**: the sequence of primers used in this study


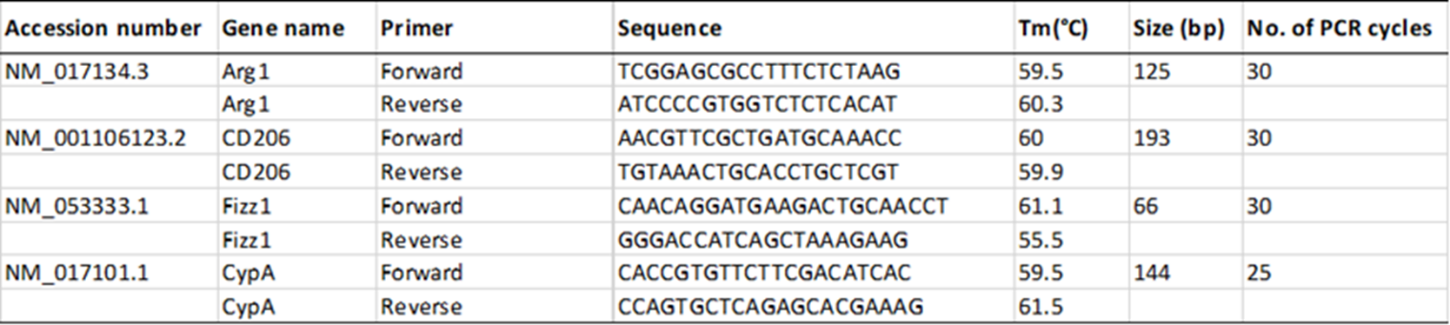

Supplement: Supplementary file 1 — Supplementary information [file 41598_2020_66977_MOESM1_ESM.docx]
